# Incidence of and Risk Factors for Infection or Colonization of Vancomycin-Resistant Enterococci in Patients in the Intensive Care Unit

**DOI:** 10.1371/journal.pone.0047297

**Published:** 2012-10-10

**Authors:** Sung-Ching Pan, Jann-Tay Wang, Yee-Chun Chen, Yin-Yin Chang, Mei-Ling Chen, Shan-Chwen Chang

**Affiliations:** 1 Department of Internal Medicine, National Taiwan University Hospital, Taipei, Taiwan; 2 Center of Infection Control, National Taiwan University Hospital, Taipei, Taiwan; 3 College of Medicine, National Taiwan University, Taipei, Taiwan; D’or Institute of Research and Education, Brazil

## Abstract

The prevalence of vancomycin-resistant enterococci (VRE) colonization or infection in the hospital setting has increased globally. Many previous studies had analysed the risk factors for acquiring VRE, based on cross-sectional studies or prevalent cases. However, the actual incidence of and risk factors for VRE remain unclear. The present study was conducted in order to clarify the incidence of and risk factors for VRE in the intensive care unit (ICU). From 1^st^ April 2008 to 31^st^ March 2009, all patients admitted to a surgical ICU (SICU) were put on active surveillance for VRE. The surveillance cultures, obtained by rectal swab, were taken on admission, weekly while staying in the SICU, and on discharge from the SICU. A total of 871 patients were screened. Among them, 34 were found to carry VRE before their admission to the SICU, and 47 acquired VRE during their stay in the SICU, five of whom developed VRE infections. The incidence of newly acquired VRE during ICU stay was 21.9 per 1000 patient-days (95% confidence interval [CI], 16.4–29.1). Using multivariate analysis by logistic regression, we found that the length of ICU stay was an independent risk factor for new acquisition of VRE. In contrast, patients with prior exposure to first-generation cephalosporin were significantly less likely to acquire VRE. Strategies to reduce the duration of ICU stay and prudent usage of broad-spectrum antibiotics are the keys to controlling VRE transmission.

## Introduction

Since 2003, vancomycin-resistant enterococci (VRE) have been one of the most important nosocomial pathogens in the United States of America (USA) [1]. The number of patients hospitalized for treatment of VRE infections doubled between 2003 and 2006, and, according to data collected from the National Nosocomial Infections Surveillance System from 1998 to 2002, VRE isolates make up 12.8% of enterococcal isolates recovered from patients in the intensive care unit (ICU), 12% of isolates recovered from non-ICU patients, and 4.7% of isolates recovered from outpatients in the USA [1]. Clinical impacts of VRE include the limited availability of drugs to treat VRE infections and the ability of VRE to transfer the genetic determinant for vancomycin resistance to other Gram-positive pathogens, such as *Staphylococcus aureus* [2]–[3]. VRE, though less virulent than other common pathogenic bacteria, such as *Staphylococcus aureus eg*, could cause severe infections in vulnerable or immunocompromised patients, e.g., bloodstream, intra-abdominal, or urinary tract infections. Despite some conflicting data, several studies have demonstrated that patients with VRE bacteraemia have a higher mortality rate than those infected with vancomycin-susceptible enterococci [4]–[6].

**Table 1:** Demographic and clinical data from 46 patients who acquired VRE in the ICU and 184 control patients.

Parameter	VRE(N = 46)	NO VRE (N = 184)	P-value
Age	66.46±13.60	66.84±14.87	0.87
Male gender, n (%)	33 (71.74)	115 (62.50)	0.24
Length of risk in ICU* (days)	13.61±13.45	8.28±8.76	0.01
**Reasons for admission to ICU, n (%)**			
Septic shock	11 (23.91)	15 (8.15)	0.0025
Respiratory failure	10 (21.74)	24 (13.04)	0.14
Heart failure	1 (2.17)	1 (0.54)	0.36
Acute myocardial injury	1 (2.22)	3 (1.63)	0.59
Acute renal failure	2 (4.35)	1 (0.54)	0.10
Post-operative care	17 (36.96)	86 (46.74)	0.23
Coronary artery bypass graft	0 (0.00)	1 (0.54)	1.00
Intracranial haemorrhage	1 (2.17)	5 (2.72)	1.00
Hollow organ peroration	4 (8.70)	24 (13.04)	0.42
Trauma	1 (2.17)	6 (3.26)	1.00
Others	8 (17.39)	26 (14.21)	0.59
**Underlying diseases**			
**Cardiovascular**	21 (45.65)	114 (61.96)	0.04
Hypertensive heart disease	0 (0.00)	0 (0.00)	-
Valvular heart disease	0 (0.00)	1 (0.54)	1.00
Coronary artery disease	3 (6.52)	22 (11.96)	0.29
Cardiomyopathy	0 (0.00)	0 (0.00)	-
Heart failure	1 (2.17)	10 (5.43)	0.70
Hypertension	17 (36.96)	99 (53.80)	0.04
Other cardiovascular diseases	5 (10.87)	33 (17.93)	0.25
**Respiratory**	6 (13.04)	22 (11.96)	0.84
Chronic obstructive pulmonary disease	4 (8.70)	9 (4.89)	0.30
Respiratory failure	1 (2.17)	2 (1.09)	0.49
Other respiratory diseases	2 (4.35)	11 (5.98)	1.00
**Hepatobiliary**	17 (36.96)	42 (22.83)	0.05
Liver cirrhosis	10 (21.74)	10 (5.43)	0.0016
HBV carrier	12 (26.09)	24 (13.04)	0.03
HCV carrier	4 (8.70)	8 (4.35)	0.26
Alcohol hepatitis	1 (2.17)	1 (0.54)	0.36
Other hepatitis	0 (0.00)	0 (0.00)	-
Cholelithiasis	2 (4.35)	2 (1.09)	0.18
Other hepatobiliary diseases	6 (13.04)	12 (6.52)	0.21
**Genitourinary**	14 (30.43)	62 (33.70)	0.67
Chronic kidney disease	7 (15.22)	32 (17.4)	0.92
Other genitourinary diseases	9 (19.57)	36 (19.57)	1.00
**Gastroenterologic disease**	17 (36.96)	50 (27.17)	0.19
**Mucocutaneous disease**	0 (0.00)	0 (0.00)	-
**Neurovascular**	4 (8.70)	28 (15.22)	0.25
Cerebrovascular accident	2 (4.35)	19 (10.33)	0.26
Degenerative disease	1 (2.17)	9 (4.89)	0.69
Other neurovascular disease	1 (2.17)	2 (1.09)	0.49
**Endocrinologic comorbidity**	11 (23.91)	77 (41.85)	0.03
Diabetes mellitus	10 (21.74)	56 (30.43)	0.24
Other endocrinologic disease	2 (4.35)	33 (17.93)	0.02
**Autoimmune**	3 (6.52)	5 (2.72)	0.20
Systemic lupus erythematosus	0 (0.00)	2 (1.09)	1.00
Rheumatic arthritis	2 (4.35)	1 (0.54)	0.10
Others	2 (4.35)	2 (1.09)	0.18
**Solid organ malignancy**	24 (52.17)	107 (58.15)	0.46
**Haematologic malignancy**	1 (2.17)	3 (1.63)	1.00
Leukaemia	0 (0.00)	2 (1.09)	1.00
Lymphoma	1 (2.17)	1 (0.54)	0.36
**Receiving immune suppressive medication**	6 (13.04)	23 (12.50)	0.92
Steroid	4 (8.70)	14 (7.61)	0.76
FK506	0 (0.00)	3 (1.63)	1.00
Cellcept	0 (0.00)	6 (3.26)	0.60
Others	4 (8.70)	12 (6.52)	0.53
**Recent operation**	32 (69.57)	148 (80.43)	0.11
Abdomen	25 (54.35)	101 (54.89)	0.95
Others	7 (15.22)	47 (25.54)	0.14
Smoking	15 (34.09)	63 (34.24)	0.99
Alcoholism	11 (25.00)	40 (21.74)	0.64
APACHE II score	23.07±7.80	20.97±6.72	0.07
**Invasive catheter use**			
Use of CVC	45 (97.83)	165 (90.66)	0.13
Use of drainage tube	32 (69.57)	134 (72.83)	0.66
Use of double lumen CVC	13 (35.14)	35 (20.00)	0.049
Use of nasogastric tube	42 (93.33)	167 (91.26)	1.00
Use of foley	41 (93.18)	171 (94.48)	0.72
Use of endotrachial tube	45 (97.83)	168 (91.30)	0.22
Antibiotic use	45 (97.83)	184 (100.00)	0.20
Penicillin	7 (15.22)	49 (26.63)	0.11
Antipseudomonal penicillin	11 (23.91)	57 (30.98)	0.35
Penicillins combined with β-lactamase inhibitors	6 (13.04)	36 (19.57)	0.31
First-generation cephalosporin	5 (10.87)	80 (43.48)	<0.0001
Second-generation cephalosporin	8 (17.39)	71 (38.59)	0.0068
Third-generation cephalosporin	14 (30.43)	46 (25.00)	0.45
Anti-pseudomonal third –generation cephalosporin	15 (32.61)	36 (19.57)	0.06
Fourth-generation cephalosporin	11 (23.91)	31 (16.85)	0.27
Carbapenem	12 (26.09)	41 (22.28)	0.58
Monobactam	0 (0.00)	1 (0.54)	1.00
Glycopeptide	14 (30.43)	39 (21.20)	0.18
Metronidazole	15 (32.61)	59 (32.07)	0.94
Aminoglycoside	5 (10.87)	39 (21.20)	0.11
Antifungal	15 (32.61)	32 (17.39)	0.02
Fluoroquinolone	4 (8.70)	19 (10.33)	1.00
Colistin	1 (2.17)	4 (2.17)	1.00
Tigecycline	1 (2.17)	7 (3.26)	1.00

* Length of risk was defined as the duration from ICU admission to the day culture revealed VRE during ICU stay for the case group and was defined as the entire duration of ICU stay for the control group.

CVC: central venous catheter.

In Taiwan, the prevalence of VRE remained low before 2007. However, according to surveillance data from the Taiwan Nosocomial Infections Surveillance program, the proportion of VRE among all enterococcal isolates causing nosocomial infections increased from 3% in 2003 to 15.5–23.6% in 2010 [7]. To control the rapid spread of multidrug resistant organisms, it is necessary to understand the risk factors for acquiring them. In previous studies, advanced age, renal and hepatic failure, haematological malignancy, underlying illness, invasive procedures and devices, gastrointestinal surgery, transplantation, proximity to another VRE-positive patient, and broad spectrum antimicrobial therapy have been identified as risk factors for VRE colonization and/or infection [8]–[11]. However, many of these prior reports were cross-sectional and enrolled prevalent cases instead of incident cases for analysis, possibly leading to biases in the study results. Therefore, we conducted the following study to identify the incidence of VRE, rather than the prevalence, and the associated risk factors for newly acquired VRE during ICU stay.

## Materials and Methods

### Ethics Statement

We followed the principles expressed in the Declaration of Helsinki. This study was approved by the Institutional Ethics Review Board of the National Taiwan University Hospital. Waived inform consent had been approved due to retrospective study design and the research involved no more than minimal risk.

### Patients

From 1^st^ April 2008 to 31^st^ March 2009, all patients admitted to the surgical ICU (SICU) at the National Taiwan University Hospital (NTUH), a major tertiary teaching hospital with a capacity of 2,200 beds located in northern Taiwan, were enrolled in this study. Surveillance cultures were obtained from all patients in the SICU as follows: 1) cultures were taken from all patients in the SICU on the first day of the study; 2) patients who were newly admitted to the SICU had surveillance cultures taken within 24 h after admission; 3) for all patients, surveillance cultures were taken every week after admission and on the day of discharge from the SICU; 4) rectal swabs were used as surveillance cultures. All swabs were sent to the infection control laboratory for bacterial culture and subsequent microbiologic studies. The culture results of clinical specimens from the enrolled patients, which were performed at the central laboratory at NTUH, were also collected. Patients positive for VRE were then put on strict contact isolation [12]. Only the first instance of VRE occurrence was considered.

### Data Collection and Definitions

This was a case-controlled study to identify risk factors for VRE. Patients who acquired VRE, either colonization or infection, during their stay in the ICU were classified as the case group. Patients who were negative for VRE during their stay in the ICU were used as the selection pool for the control group. The control patients were then randomly sampled from this selection pool using a computer-generated random digital number table with a 4∶1 ratio to the case group. A standardized case report form was used to collect demographic, clinical, and microbiological data from both case and control patients. The demographic data included patients’ ages and sexes. Clinical data included dates of ICU admission and discharge, length of stay in the ICU, duration of risk to acquire VRE (length of stay in ICU before acquiring VRE; for those who were not found to carry VRE for the entire ICU stay, we used the duration of the whole ICU stay for this variable), APACHE II score upon admission to the ICU, underlying diseases, recent operations, invasive therapeutic procedures, and prior usage of antimicrobial agents. The APACHE II scores were divided into low and high groups, using a cut-off point of 17, as suggested by previous reports [13]. Prior usage of antibiotics was defined as antibiotics used within 15 days before acquisition of VRE (for those who were positive for VRE) or during the period from 15 days before SICU admission to the time of discharge from the SICU (for those who were negative for VRE) [14]. Recent operation was defined as: 1) a simple procedure performed under local anaesthesia within seven days prior to acquisition of VRE (for those who were positive for VRE) or during the period from seven days before ICU admission to the time of discharge from the SICU (for those who were negative for VRE); 2) an operation performed under general anaesthesia within 30 days prior to acquisition of VRE (for those who were positive for VRE) or during the period from 30 days before ICU admission to the time of discharge from the ICU (for those who were negative for VRE) [15]. All patients’ data had been processed before analysis so that the patients’ identities could not be discerned.

**Table 2:** Univariate analysis of risk factors for acquiring VRE during ICU stay.

Parameter	OR (95% CI)	*P*-value
Age	1.00 (0.98–1.02)	0.87
Sex	1.52 (0.75–3.09)	0.24
Length of risk	1.04 (1.02–1.07)	0.003
**Reasons for admission to ICU**		
Septic shock	3.54 (1.50–8.36)	0.004
Respiratory failure	1.85 (0.81–4.21)	0.14
Heart failure	4.07 (0.25–66.27)	0.32
Acute myocardial injury	1.37 (0.14–13.50)	0.79
Acute renal failure	8.32 (0.74–93.82)	0.09
Post-operative care	0.67 (0.34–1.30)	0.23
Coronary artery bypass graft	–	–
Intracranial haemorrhage	0.80 (0.09–6.98)	0.84
Hollow organ perforation	0.63 (0.21–1.93)	0.42
Trauma	0.66 (0.08–5.62)	0.70
Others	1.27 (0.53–3.03)	0.59
**Underlying diseases**		
Cardiovascular	0.51 (0.27–0.99)	0.047
Respiratory	1.10 (0.42–2.90)	0.84
Hepatobiliary	1.98 (0.99–3.95)	**0.05**
Genitourinary	0.86 (0.43–1.73)	0.67
Gastroenterologic	1.57 (0.80–3.11)	0.19
Mucocutaneous	–	–
Neurovascular	0.53 (0.18–1.60)	0.26
Endocrinologic	0.44 (0.21–0.91)	0.03
Autoimmune	2.50 (0.58–10.86)	0.22
Solid organ malignancy	0.79 (0.41–1.50)	0.46
Haematological malignancy	1.34 (0.14–13.20)	0.80
Immune suppressive medication	1.05 (0.40–2.75)	0.92
Recent operation	0.56 (0.27–1.15)	0.11
Smoke	0.99 (0.50–1.99)	0.99
Alcoholism	1.20 (0.56–2.58)	0.64
APACHE II score	1.04 (1.00–1.09)	0.07
**Invasive catheter use**		
Central venous catheter	4.63 (0.60–35.78)	0.14
Drainage tube	0.85 (0.42–1.73)	0.66
Double lumen	2.17 (1.00–4.68)	0.049
Nasogastric tube	1.34 (0.37–4.82)	0.65
Foley	0.80 (0.21–3.04)	0.74
Endotracheal tube	4.29 (0.55–33.18)	0.22
**Antibiotic usage**		
Penicillin	0.50 (0.21–1.18)	0.11
Anti-pseudomonal penicillin	0.70 (0.33–1.48)	0.35
Penicillins combined with β-lactamase inhibitors	0.62 (0.24–1.57)	0.31
First-generation cephalosporin	0.16 (0.06–0.42)	0.0002
Second-generation cephalosporin	0.34 (0.15–0.76)	0.009
Third-generation cephalosporin	1.31 (0.65–2.67)	0.45
Antipseudomonal third-generation cephalosporin	1.99 (0.97–4.07)	0.06
Fourth-generation cephalosporin	1.55 (0.71–3.38)	0.27
Carbapenem	1.23 (0.59–2.59)	0.58
Monobactam	–	–
Glycopeptide	1.62 (0.79–3.34)	0.19
Metronidazole	1.03 (0.51–2.04)	0.94
Aminoglycoside	0.45 (0.17–1.22)	0.12
Antifungal	2.30 (1.11–4.75)	0.02
Fluoroquinolone	0.83 (0.27–2.56)	0.74
Colistin	1.00 (0.11–9.17)	1.00
Tigecycline	0.66 (0.08–5.62)	0.70

* If any cell in the analysis was 0, then the calculation of odd ratios could not be performed.

### Bacterial Culture and Identification of VRE

VRE isolates were screened by bile esculin azide broth containing 8 µg/mL vancomycin (BEAV) and chromogenic agar medium (chromID VRE; BioMérieux, Marcyl’Etoile, France). Colonies on the chromID VRE plates with purple or green pigmentation were presumptively identified as vancomycin-resistant *Enterococcus faecium* (VRE*Fium*) or *E. faecalis* (VRE*Flis*), respectively. If necessary, VRE colonies were then subcultured onto Trypticase soy agar plates supplemented with 5% sheep blood (BBL) for confirmatory identification using the Vitek2 System (BioMérieux, Marcyl’Etoile, France).

The electrokaryotypes of VRE isolates digested by the *Sma*I restriction enzyme were analysed by pulsed-field gel electrophoresis (PFGE) according to previously described methods [12], [16]. PFGE patterns were determined using the Pearson product-moment correlation coefficient, with the Gel Compare II software package (Applied Maths BVBA, Austin Texas). Unweighted-pair group method using average linkages (UPGMA) dendrograms were constructed from these data. Isolates that exhibited similarities greater than 86.7% of their banding patterns were considered to belong to the same pulsotypes [17].

**Table 3:** Multivariate analysis of risk factors for acquiring VRE during ICU stay.

Parameter	OR (95% CI)	P-value
Length of risk	1.03 (1.004–1.065)	0.03
Prior use of first-generationcephalosporin	0.18 (0.07–0.48)	0.0007

### Drug Susceptibility Tests

In vitro susceptibilities of the isolates to penicillin G, ampicillin, gentamicin, teicoplanin, tetracycline, and ciprofloxacin were determined by the disk diffusion method defined by the CLSI [18]. If necessary, they were confirmed using the Vitek2 System (BioMérieux, Marcyl’Etoile, France).

### Statistics

Continuous variables were presented as means ± standard deviations (SDs) and were compared using the Student’s *t* test. Categorical variables were compared using a chi-squared test or Fisher’s exact test if the expected values were below 5. The prevalence of VRE and incidence of newly acquired VRE during ICU stay were determined. To analyse the risk factors for acquiring VRE during ICU stay, we used logistic regression to compare patients with newly acquired VRE and those without VRE colonization/infection during ICU stay. All parameters were initially tested by univariate analysis; those with *P*-values less than 0.1 and those that were biologically meaningful were used for multivariate analysis. However, parameters with colinearity, tested by correlation matrices, were not simultaneously considered in the final model. In multivariate analysis, stepwise model comparison was used to determine the best model. Statistical analyses were performed using SAS 9.1.3 (SAS Institute Inc., Cary, NC, USA). All tests were 2-tailed and *P*-values less than 0.05 were considered statistically significant.

## Results

During the study period, a total of 1874 specimens were collected from 871 patients. Among these patients, 51 were carriers of VRE before they were admitted to the ICU, 47 were found to acquire VRE during their stay in the ICU, and 773 were negative for VRE during their stay in the ICU. Among the 47 patients who acquired VRE during their ICU stays, five had VRE infections (three urinary tract infections [UTIs], one had a blood stream infection [BSI], and one had a UTI combined with a BSI and skin and soft tissue infection). The others were colonizers. Overall, 2149 patient-days at risk were observed. Therefore, the incidence of acquiring VRE during ICU stay was 21.9 per 1000 patient-days (95% CI, 16.4–29.1 per 1000 patient-days). The overall prevalence of VRE among patients in the ICU was 11.3% (95% CI, 0–13.4%).

The clinical data for one of the 47 patients who acquired VRE while in ICU was not available. Therefore, only 46 patients were included in the final case group. Among the 773 patients without VRE isolated as the selection pool for the control group, 184 patients were randomly selected as control patients. The epidemiological and clinical data from the 46 case patients and 184 control patients are listed in Table 1.

Our data demonstrated that patients who acquired VRE had a longer duration of ICU stay prior to acquiring VRE, were more likely to be admitted to the ICU for septic shock, were less likely to have underlying cardiovascular and endocrinologic diseases, were more likely to have underling liver cirrhosis or hepatitis B virus (HBV) infection, were more likely to be implanted with a double lumen catheter, were more likely to receive antifungal treatment, and were less likely to receive first- and second-generation cephalosporins 15 days before acquiring VRE (Table 2).

Multivariate analysis showed that length of ICU stay was an independent risk factor for new acquisition of VRE during ICU stay (OR, 1.034; 95% CI, 1.004–1.065; P = 0.03). On the other hand, prior usage of first generation cephalosporin was a protective factor against new acquisition of VRE (OR, 0.181; 95% CI, 0.07–0.48; P = 0.0007; Table 3).

### VRE Isolates

All VRE isolates were VRE *faecium*, and 9.9% were susceptible to gentamicin, 38.3% were susceptible to tetracycline, and 19.8% were susceptible to teicoplanin; all were resistant to ciprofloxacin. There was no statistical difference in susceptibility to antibiotics among isolates collected from patients with previous colonization before admission to the ICU or new acquisition of VRE after ICU admission (Table 4).

**Figure 1:**
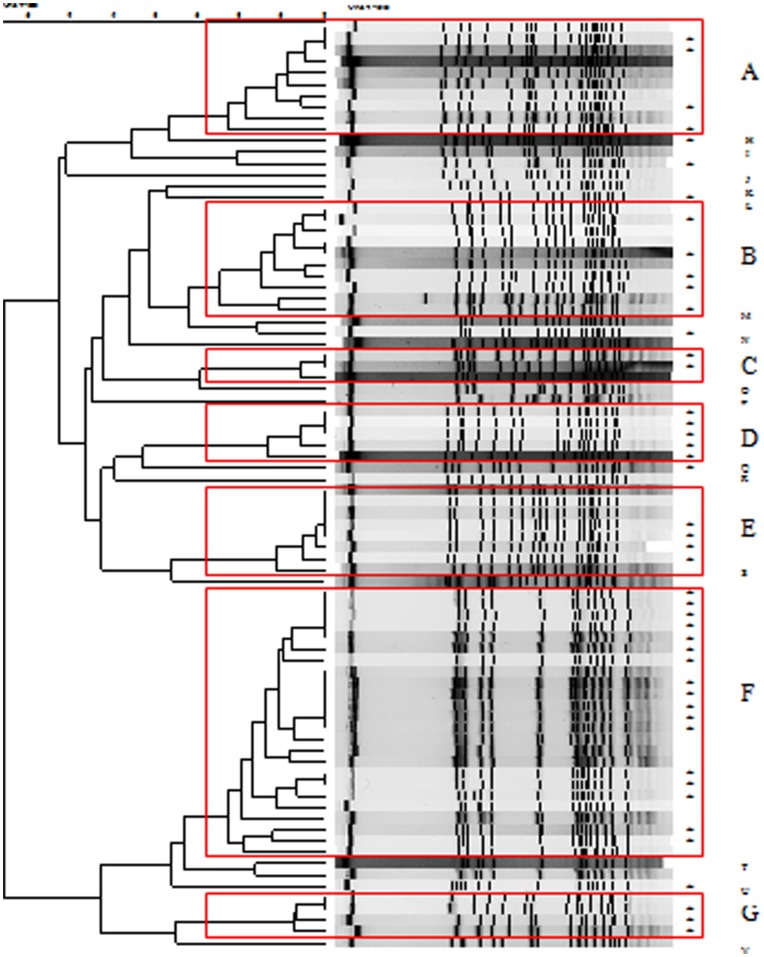
Pulse-field gel electrophoresis of 82 isolates of vancomycin-resistant enterococcus. Seven major pulsotypes (A–G) and 15 minor pulsotypes (H–V) were identified. *, incidence case acquired after ICU admission, all others were prevalent cases acquired before ICU admission.

Among the 81 isolates available for PFGE, we identified seven major pulsotypes (A–G) and 15 minor pulsotypes (H–V; Figure 1). For pulsotype A, there were six isolates from control patients who acquired VRE before ICU admission (prevalent isolates) and four isolates from patients who acquired VRE during ICU stay (incidence isolates). For pulsotypes B–G, there were five, one, zero, three, seven, and one prevalent isolates and five, two, six, four, 17, and three incidence isolates, respectively.

## Discussion

In this study, the incidence of newly acquired VRE was 21.9 per 1000 patient-days in an ICU setting. The major risk factor for VRE acquisition was prolonged ICU stay. In contrast, prior usage of first-generation cephalosporin was negatively associated with VRE colonization.

Previously, several studies had investigated the epidemiology of VRE in high-risk patients, such as ICU patients [19]–[23]. However, most studies reported prevalence data, and only a few reported data describing the incidence of VRE acquisition. Because VRE colonization may persist for years, prevalence data may mainly reflect the duration of colonization [24]–[25]. Thus, the incidence of newly acquired VRE may more accurately present the dynamic changes in the resistance burden of clinical settings, and infection control or antibiotics policies can be adjusted accordingly through serial follow-up. Through a national nosocomial infection surveillance system, Kohlenberg *et al*. reported that the mean incidence of VRE cases in the ICU in Germany during 2005–2006 was 0.29 cases per 1000 patient-days, with significant regional differences [19]. Warren et al reported a VRE incidence of 27 cases per 1000 patient-ICU days at a medical ICU in the USA [20]. Our data were similar to the incidence of VRE in the USA and the resistance burden may increase gradually. Since the prevalence of MRSA among ICU patients in Taiwan is also high, we should be attentive to the possible coexistence of MRSA and VRE, leading to VRSA in this population [14]. Further surveillance is warranted.

**Table 4:** Drug susceptibility tests among patients with previous colonization and newly acquired VRE.

	All	Previous colonization	Newly –acquired	P-value*
	(N = 81)	(N = 34)	(N = 47)	
Penicillin G	0/81 (0%)	0/34 (0%)	0/47 (0%)	–
Ampicillin	0/81 (0%)	0/34 (0%)	0/47 (0%)	–
Gentamicin	8/81 (9.9%)	4/34 (11.8%)	4/47 (1.4%)	0.90
Tetracycline	31/81 (38.3%)	15/34 (44.1%)	16/47 (34.0%)	0.36
Ciprofloxacin	0/81 (0%)	0/34 (0%)	0/47 (0%)	–
Teicoplanin	16/81 (19.8%)	8/34 (23.5%)	8/47 (17.0%)	0.47

* Comparing previously colonized VRE strains with newly acquired strains.

Risk factor analysis for VRE acquisition is important in preventing and controlling the burden of VRE in the clinical setting, and this has been discussed in many previous studies [20]–[23], [26]. However, the majority of these studies have focused on the prevalence of VRE, rather than the incidence of newly acquired VRE after ICU admission. Thus, these previous risk factor analyses may be biased because the temporality between VRE acquisition and the presence of variables cannot be clarified. Our study compared patients with newly acquired VRE and patients without VRE during their ICU stay. Under such a study design, the temporality was much clearer. Our results showed that patients who received first-generation cephalosporin were at a lower risk of VRE acquisition. Further analysis revealed that patients who received first-generation cephalosporin had a lower chance of receiving second- or third-generation cephalosporin, third-generation cephalosporin with antipseudomonal effects, carbapenem, metronidazole, antifungals, fluoroquinolone, and tigecycline. In this way, exposure to first-generation cephalosporin can be used as a surrogate marker to indicate whether patients had prior exposure to broad-spectrum antibiotics and therefore were at a lower risk of VRE acquisition due to reduced selective pressure from prior antibiotics.

The other independent risk factor for VRE acquisition in the ICU was longer ICU stay. Each day in the ICU increased the risk of acquiring VRE by 1.03 times. This result was comparable to most previous studies [20], [22], [23]. Longer ICU stays can indicate a greater chance to receive antibiotics (selective pressure) and also a longer time to be exposed to possible pathogen transmission. According to the PFGE analysis, there were seven major pulsotypes noted during the study period. Most pulsotypes consisted of both incident and prevalent isolates, except pulsotype D, which contained incident isolates only. This implies that the VRE acquisition within the ICU may mainly result from cross transmission of prevalent isolates. Although active surveillance cultures were performed, quarantine of patients with VRE would occur only after the culture results were available, usually 3–5 days after sampling. If the patient carried VRE, then transmission could occur before contact isolation was initiated. Adherence to infection control precautions by ICU staff may also affect possible VRE transmission. Other distinct strains may come from intrinsic enterococcus, selected for under selective pressure. Thus, infection control policy, in conjunction with antibiotic stewardship, is important to combat VRE transmission in these high-risk patients.

Disease severity and underlying host conditions were not found to be important risks for acquiring VRE in our study. This result is consistent with a previous study in an adult bone marrow and stem cell transplant unit (BMT), in which underlying conditions, such as previous transplantation, recent transplantation, and no transplantation did not affect the risk for new acquisition of VRE while hospitalized in the BMT [27]. This phenomenon may only reflect the selection of the control group. When all the study participants had similar underlying conditions and disease severities, the importance of disease severity and underlying condition would not be significantly different.

The major limitation of this study is that it was conducted in the ICU; thus, the results may not be representative of other low-risk sections of the hospital. However, since patients in the ICU carry the highest risk for acquiring VRE, which in turn leads to increased risk of mortality, mobility, and medical costs, our study results are still meaningful and relevant to important clinical issues [9], [10], [28], [29].

The other limitation is that some cases of incident VRE may come from episodes of VRE outbreaks, affecting the risk factor analysis. However, since the antibiotics used and the duration of ICU stays remained significantly associated with VRE acquisition, these two risk factors appeared to be robust.

In conclusion, we studied the incidence of and risk factors for acquiring VRE in the ICU. Appropriate use of antibiotics to reduce the selective pressure caused by broad-spectrum antibiotics, adherence to infection control measures, and shortening the duration of ICU stay may help to control the spread of VRE in the ICU setting.
